# *Saccharomyces cerevisiae* and Caffeine Implications on the Eukaryotic Cell

**DOI:** 10.3390/nu12082440

**Published:** 2020-08-13

**Authors:** Lavinia Liliana Ruta, Ileana Cornelia Farcasanu

**Affiliations:** Department of Organic Chemistry, Biochemistry and Catalysis, Faculty of Chemistry, University of Bucharest, Sos. Panduri 90-92, 050663 Bucharest, Romania; lavinia.ruta@chimie.unibuc.ro

**Keywords:** caffeine, *Saccharomyces cerevisiae*, irradiation, DNA damage, TOR, signaling, lifespan

## Abstract

Caffeine–a methylxanthine analogue of the purine bases adenine and guanine–is by far the most consumed neuro-stimulant, being the active principle of widely consumed beverages such as coffee, tea, hot chocolate, and cola. While the best-known action of caffeine is to prevent sleepiness by blocking the adenosine receptors, caffeine exerts a pleiotropic effect on cells, which lead to the activation or inhibition of various cell integrity pathways. The aim of this review is to present the main studies set to investigate the effects of caffeine on cells using the model eukaryotic microorganism *Saccharomyces cerevisiae*, highlighting the caffeine synergy with external cell stressors, such as irradiation or exposure to various chemical hazards, including cigarette smoke or chemical carcinogens. The review also focuses on the importance of caffeine-related yeast phenotypes used to resolve molecular mechanisms involved in cell signaling through conserved pathways, such as target of rapamycin (TOR) signaling, Pkc1-Mpk1 mitogen activated protein kinase (MAPK) cascade, or Ras/cAMP protein kinase A (PKA) pathway.

## 1. Introduction

Caffeine (1,3,7-trimethylxanthine) is the best-known chemical constituent of coffee and one of the most widely consumed and socially accepted natural stimulants. As an important constituent of coffee, but also of other largely-consumed beverages such as tea, chocolate, and cola-like drinks, caffeine is by far the most ingested methylxanthine, along with the less representative theophylline (1,3-dimethylxanthine, encountered in tea) and theobromine (1,7-dimethylxanthine, mostly found in cocoa). Caffeine is also widely used as an important ingredient of various medicine and non-prescription drugs (used against headaches, common cold, or as appetite suppressants), sports and energy drinks, nutritional supplements, and cosmetics. The scientific literature dealing with the biological effects of caffeine is vast, revealing a large amount of evidence on both the beneficial and deleterious effects, on indications and contraindications, on adverse effects and toxicity, etc. The action of caffeine at the cellular level has been intensively investigated, and there are three fundamental mechanisms which are universally recognized: intracellular mobilization of calcium, inhibition of phosphodiesterases, and antagonism at the level of adenosine receptors [1].

**Figure 1:**
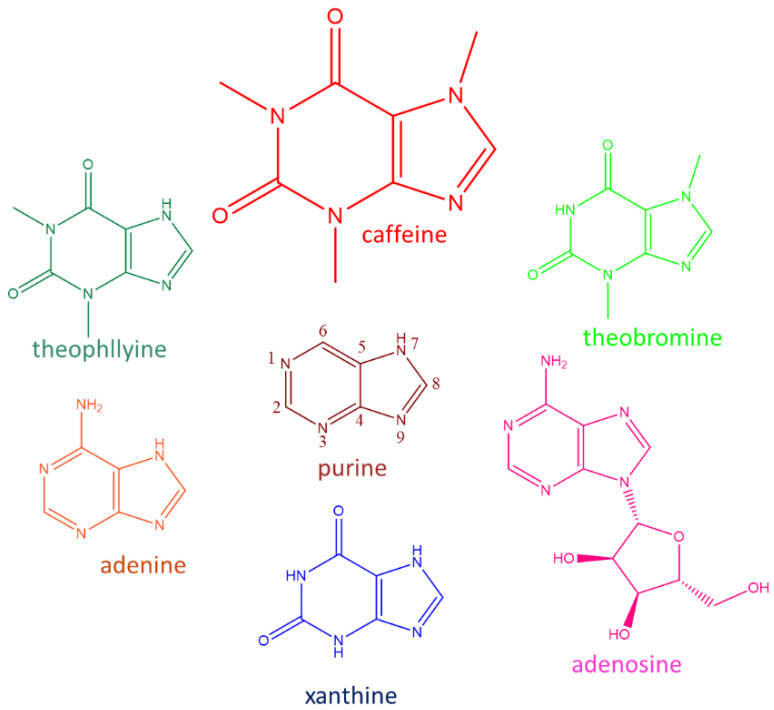
Chemical structures of some purines chemically related to caffeine.

Caffeine belongs to the purine alkaloid family closely linked with the bases adenine and guanine (Figure 1), which are fundamental components of nucleosides, nucleotides, and the nucleic acids [2]. Caffeine is a low-affinity adenosine and ATP analogue which interacts with a number of cellular processes, including cell growth, DNA metabolism, and cell cycle progression [3].

In this review, we present some of the studies set to unravel the caffeine mechanisms of action using *Saccharomyces cerevisiae* as a model for the eukaryotic cell. A model organism is used in scientific research for various reasons: simplification of the biological context, overcoming ethical and experimental constraints, elimination of redundancies, the establishment of a framework for development and optimization of analytical methods, etc. Importantly, a model organism has to be representative of a larger class of living beings [4]. *S. cerevisiae,* a relatively simple unicellular eukaryote, has emerged as a versatile and robust model organism to study the fundamental factors that determine eukaryotic cell biology [5]. *S. cerevisiae* is most utilized by the research community due to its amenability to genetic studies, comprehensive genome annotation [6], and a high degree of homology of essential cellular organization and metabolism with higher eukaryotes [7]. Additionally, *S. cerevisiae* is an invaluable tool in genomic studies, resistance profiling, metabolome studies, and metabolic engineering [8,9,10,11,12]. *S. cerevisiae* offers insights into the complex mechanisms underlying the sensing and response to the external conditions, including exposure to a plethora of synthetic and natural chemical compounds, such as caffeine. *S. cerevisiae* is generally responsive to caffeine, as it was uncovered that this substance affects yeast cell growth and morphology, DNA repair mechanisms, intracellular calcium homeostasis, and cell cycle progression [13].

This paper provides an overview on the studies that used *S. cerevisiae* to unravel some potential effects of caffeine on the eukaryotic cells, with a focus on caffeine transport in yeast cells, caffeine influence on cells exposed to irradiation, caffeine interaction with target of rapamycin (TOR) and cell wall integrity pathways, and caffeine influence on the lifespan of the cells. 

## 2. Caffeine: Transport and Toxicity in *S. cerevisiae*

The effects of caffeine on cells are pleiotropic, causing delays to cell cycle progression; changes in cell morphology; and in high doses, cytotoxicity. Due to structural similarity to nucleotides (Figure 1), it has been considered that caffeine taken up by the cells could affect DNA replication and/or transcription [13]. Caffeine uptake by *S. cerevisiae* cells has not been investigated in detail. Being non-essential, it is expected that caffeine would be carried into the cell by a non-specific transporter, such as purine permease. In *S. cerevisiae*, *FCY2* encodes for a purine-cytosine permease, which mediates purine (adenine, guanine, and hypoxanthine) and cytosine accumulation [14]. Fcy2 may also translocate caffeine into the yeast cell, as it was shown that *fcy2Δ* knock-out strain cannot accumulate caffeine from the medium [15]. In the yeast genome, two more purine-cytosine permease encoding genes are annotated, *FCY21* and *FCY22*, which belong to the same family as *FCY2*. Nevertheless, despite of the nucleotide sequence similarity, neither *FCY21* nor *FCY22* can complement *FCY2* absence [16], and the role of Fcy21 or Fcy22 in the eventual caffeine accumulation has not been specifically investigated.

Caffeine efflux into the extracellular space is better understood, and it is ensured by two ATP-binding cassette (ABC)-transporters responsible for the multidrug resistance in yeast, i.e., Snq2 and Pdr5. In fact, Snq2 was firstly described as the transporter responsible for caffeine detoxification, when *SNQ2* was identified as a caffeine-resistance gene by screening a genomic library of *S. cerevisiae* in a multicopy vector. Multicopy of *PDR5* also conferred resistance to caffeine but to a lower extent compared to *SNQ2* [17]. Pdr5 is also a plasma membrane ABC transporter and a functional homolog of Snq2. Investigation of the functional roles of Snq2 and Pdr5 demonstrated that Snq2 and Pdr5 mediate caffeine efflux (and subsequently caffeine resistance) in *S. cerevisiae* cells [17]. Using evolutionary engineering and molecular characterization of a caffeine-resistant *S. cerevisiae* strain, it was found that caffeine resistance could be gained generally by overexpression of pleiotropic drug resistance genes. The study identified a mutation in *PDR5* but also in *PRD1*, which encodes the transcription factor which regulates *PDR5* and *SNQ2* expression, indicating that resistance to caffeine can be correlated with an efficient and active system of extrusion from the cell [18]. An ABC-transporter gene *BFR1* from *Schizosaccharomyces pombe* was expressed into *S. cerevisiae*, resulting in enhanced caffeine resistance, suggesting that ABC-transporters can be an efficient way to reduce caffeine toxicity in heterologous systems [19]. In mammals, caffeine detoxification is mediated by P450 enzyme [20] and while no specific transporter has been associated with caffeine cellular export, multidrug resistance transporters cannot be excluded. In this line of evidence, caffeine was often used as a pharmacological substrate when studying ABC drug transport characteristics of mammalian cell lines, especially in cocktail approaches [21,22,23,24].

Caffeine resistance was also acquired in *S. cerevisiae* by overexpression of *HSE1* (encoding a subunit of the endosomal Vps27p-Hse1p complex required for sorting of ubiquitinated membrane proteins into intralumenal vesicles prior to vacuolar degradation, as well as for recycling of Golgi proteins and formation of lumenal membranes [25]), *RTS3* (encoding a putative component of the protein phosphatase type 2A complex, [26]), and *SDS23* and *SDS24* (both encoding proteins involved in cell separation during budding [27,28]). None of these genes encodes a transporter, and the deletion of any one of these genes resulted only in mild caffeine sensitivity; nevertheless, the combination of multiple deletions strongly sensitized the yeast cells to caffeine, suggesting the multiple effects that caffeine exerts on yeast cells [29].

The pleiotropic effect of caffeine on *S. cerevisiae* can be used to develop a model to study the toxic effects of various substances [30]. It was found that caffeine toxicity is enhanced in yeast cells following exposure to cigarette smoke and that yeast efflux transporters are targets of cigarette smoke chemicals, suggesting once more that associating caffeine-rich products with smoking is not recommended [31]. In line with habitual behavior studies and considering that most of the caffeine beverages are consumed hot (tea, coffee, chocolate), it was revealed that associating caffeine exposure with hyperthermia had an increased mutagenic effect on *S. cerevisiae* cells when pure caffeine was used [32]. Caffeine was also shown to reduce the ozone-survival of the wild-type and the *rad1* and *rad6* mutants of *S. cerevisiae*, whereas no effect was observed in the *rad52* mutant [33]. The interaction between caffeine and *S. cerevisiae* has been also used as a model system to explore the toxicity of the antitumoral agent 1,3-bis(2-chloroethyl)-1-nitrosourea (BCNU), when caffeine showed some effect in enhancing BCNU toxicity by decreasing both the mutagenic and the recombinogenic potential of the drug [34]. A similar system was used to explore the potency of the Topoisomerase II poison N-[2-dimethylamino)ethyl]acridine-4-carboxamide (DACA), whose toxicity on yeast cells was slightly decreased by caffeine [35].

An exogenous substance with biological activity is expected to exert some effect at the plasma membrane level, and the ability of caffeine to block calcium entry into the yeast cell is one of such effects [36]. Caffeine was found to act upon the yeast plasma membrane by effectively inhibiting the uptake of extracellular calcium induced by amiodarone but to be only moderately effective in inhibiting the amiodarone-induced release of calcium from intracellular stores, indicating that caffeine effectively blocks the uptake of extracellular calcium but does not completely eliminate the release of calcium from intracellular stores [37].

## 3. Caffeine: Between Radio-Protector and Radio-Sensitizer

*S. cerevisiae* cells is a suitable organism to study the effect of radiation upon the eukaryotic cell and also a convenient platform to identify chemicals that alter this interaction. Some early studies suggested that caffeine potentiates the biological effects of radiation and chemical mutagens in a variety of organisms, including *S. cerevisiae* [38].

### 3.1. UV Irradiation

*S. cerevisiae* cells treated with caffeine show a significant increase in radio-sensitivity to various UV doses [39], and it was reported that caffeine had a synergic effect on sensitizing the UV irradiated cells in both haploid and diploid strains [40]. These early studies incriminated caffeine as an inhibitor of DNA repair mechanisms but without relating the caffeine sensitivity with the DNA repair pathways. In subsequent studies, a pronounced inhibitory effect of caffeine on the Rad54-dependent repair of UV-irradiation damage was reported, an inhibition that was strongly dependent on the concentration of caffeine [41]. Later, other research groups confirmed the dose-dependent inhibitory effect of caffeine on the Rad54-dependent repair of UV-irradiation damage [42,43].

Caffeine was also shown to inhibit some DNA repair mechanisms, reducing the generation of *cdc^+^* colonies under UV irradiation. In *S. cerevisiae,* the *CDC8* gene encodes for a thymidylate kinase involved in DNA replication, also required when UV irradiation induces gene conversion and gene mutation events and induction of *cdc^+^* colonies. Inhibition of DNA replication by caffeine diminishes the formation of *cdc^+^* colonies, indicating that the latter arises as a result of errors in DNA replication [44].

### 3.2. γ-Irradiation

The effect of caffeine on irradiated *S. cerevisiae* cells was also studied for γ-irradiation. A slight caffeine-sensitizing effect was found for the *rad5l* and *rad54* mutants, which show defects in the repair of X-ray induced damage [45]. The results suggested that caffeine enhanced radiation-induced cell killing and that the caffeine-sensitive process involved in the repair of γ-ray-induced lesions interfered with a recombinational repair mechanism occurring in cells in S or G2 phase [41].

The DNA damage induced by γ-irradiation of *S. cerevisiae* cells was also analyzed by a single-cell gel electrophoresis of spheroplasts. By monitoring the γ-radiation-induced DNA damage, repair, and radioprotection, this study indicated a radioprotective effect of caffeine in a dose-dependent manner [46].

The *S. cerevisiae* cells have two different physiological states, aerobic and anaerobic. Cells anaerobically grown do not have functionally active mitochondria, and the energy is generated only through glycolysis. It was shown that caffeine acted like a radioprotector against γ-radiation only in the case of yeast cells grown aerobically, in the presence of oxygen. The radioprotection offered to aerobically-grown cells did not influence the recovery process through biosynthetic reparatory ways, as caffeine did not influence the DNA repair process directly. Rather, the caffeine radioprotective phenotype observed involved scavenging of the reactive oxygen species produced by irradiation [47]. In contrast, caffeine acted as a radio-sensitizer for anaerobically grown cells [47].

The effect of caffeine was also monitored in relation with the mutagenic action of ^60^Co-generated γ-radiation or of 4-nitroquinoline 1-oxide (4-NQO) exposure. The results indicated that caffeine decreased γ-radiation-induced gene conversion frequencies. In contrast, caffeine was found to increase the induced gene conversion frequency in cells treated with 4-NQO, suggesting that the repair processes following γ-irradiation or 4-NQO treatment involve different pathways [48].

## 4. Caffeine and Cell Response to DNA Damage

**Figure 2:**
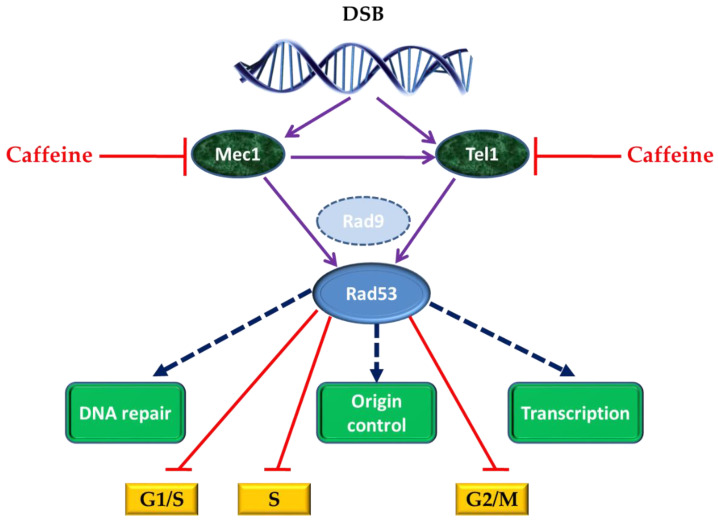
Activation of effector kinases by DNA damage in *Saccharomyces cerevisiae* cells. The central components are two redundant kinases: Mec1 (Mitosis entry checkpoint 1; ATR in mammals) and Tel1. (Telomere maintenance 1; ATM in mammals). Mec1 is hyperactivated in response to different DNA injuries and is essential for cell viability; Tel1 is activated primarily by double-strand breaks (DSBs), and its loss is not lethal in yeast. Mec1/Tel1 activate the effector kinase Rad53. In G2 phase, Rad53 activation is mediated by Rad9, in response to DNA damage. Crosstalk between Mec1 and Tel1 can occur if stalled replication forks collapse since they can generate DSBs. Rad53 inhibits G1/S, Sphase and G2/M cell cycle transitions. Adapted after [51,52].

Irradiation is often linked to DNA damage events; therefore, it was natural to investigate the effect of caffeine on cell response to DNA damage. In yeast cells, caffeine was shown to inhibit some checkpoint kinases involved in DNA double-strand breaks (DSB) repair. DSB are highly deleterious events that may lead to chromosomal abnormalities, cell death, and cancer, and repair of chromosome breaks occurs by several highly conserved pathways [49]. In *S. cerevisiae*, the response to DSB is controlled by DNA damage checkpoint signal-transduction pathways, which include the redundant protein kinases Mec1 and Tel1, members of the family of phosphatidyl inositol 3 (PI3) kinases [50,51,52], which are targets of caffeine-induced inhibition. To cope with DNA damage, Mec1/Tel1 and their downstream target kinase Rad53 regulate various cell cycle events (Figure 2). These responses allow enough time for DSB repair and ultimately for mitosis prevention in the presence of a broken chromosome.

Interaction between yeast cells and caffeine was used to demonstrate that Mec1/Tel1-dependent intra-S-phase checkpoint activation inhibits Rad52 foci formation, which occurs as a response to replication forks collapsing [53]. Induction of the intra-S-phase checkpoint by hydroxyurea (HU) inhibits Rad52 focus formation in response to ionizing radiation. This inhibition is dependent upon Mec1/Tel1 kinase activity, as HU-treated cells form Rad52 foci in the presence of the PI3 kinase inhibitor caffeine [54].

Upon activation, Mec1 and Tel1 also act directly on chromatin by phosphorylating histone H2A on seryl-129 residue to yield H2A-S129 [55]. When caffeine was used to inhibit Mec1 and Tel1 after DSB induction, it was observed that prolonged phosphorylation of H2A-S129 did not require continuous Mec1 and Tel1 activity and that caffeine treatment could affect homologous recombination also independently of Mec1 and Tel1 inhibition, by interfering with the 5′ to 3′ end resection of the DSB [56]. As similar effects of caffeine treatment were observed on irradiated HeLa cells, the potential of caffeine as a DNA damage-sensitizing agent in cancer cells is considered high, because the caffeine treatment targets one of the earliest steps in homologous recombination, independently of ATM/ATR inhibition (the PI3 kinase in mammalian cells corresponding to Mec1 and Tel1 kinases from the budding yeast) [57].

In eukaryotes, DNA damage triggers the DNA damage checkpoint, causing cells to become blocked in cell cycle progression (Figure 2). In *S. cerevisiae*, even the presence of a single DSB produces G2/M arrest, before anaphase [56]. Sometimes cells with irreparable DNA damage can escape arrest, by adaptation after a long checkpoint-mediated delay; this adaptation depends on the extent of DNA damage [57]. Srs2 is a DNA helicase and a DNA-dependent ATPase with a role in DNA repair and checkpoint recovery, and it was reported that caffeine can reverse the permanent pre-anaphase arrest of *srs2*Δ cells, supporting the idea that caffeine has the ability to override DNA damage checkpoints. Even though the cells lacking Srs2p helicase apparently completed DNA repair after caffeine treatment, the cells failed to recover, proving that Srs2p is required to turn off the DNA damage checkpoint. It was observed that inactivation of the checkpoint restores the viability of most *srs2*Δ cells, indicating that the cause of lethality of these mutant cells is the incapacity to turn off the checkpoint after the completion of DNA repair [58].

Another kinase targeted by caffeine in yeast is Kin3 kinase. In *S. cerevisiae*, *KIN3* was identified as a gene that encodes for a structural homolog of NIMA serine-threonine kinase required in *Aspergillus nidulans* for DNA damage response and in the regulation of G2/M phase progression [59,60]. *S. cerevisiae* cells that were either deleted for *KIN3* or were overexpressing it had no detectable growth phenotypes, but it was noticed that caffeine abolished *KIN3* expression induced by genotoxic agents, such as methyl methanesulfonate (MMS), cisplatin and doxorubicin, indicating that Kin3-activating signal is mediated by the caffeine-sensitive pathways. As caffeine can inhibit the DNA damage checkpoint transducers Mec1 and Tel1 [54,61], it was concluded that Kin3 can play a role in Tel1/Mec1-dependent pathway activation induced after the genotoxic stress [62].

Topoisomerases are highly conserved proteins, required for many aspects of DNA metabolism. In yeast, DNA Topoisomerase III is encoded by gene *TOP3*, whose deletion in *S. cerevisiae* causes hyperrecombination, meiotic defects, sensitivity to genotoxic agents, and poor growth due to accumulation of S/G2 DNA [63]. In a comparative analysis over the effects of caffeine on a cell culture overexpressing *TOP3* after exposure to mutagen MMS, it was observed that caffeine-treated cells successfully traverse S phase, while caffeine non-treated cells failed to show any significant recovery and remained with a mid-S DNA content, suggesting that a persistent checkpoint-mediated cell cycle delay leads to the impaired S-phase progression that can be overridden by the addition of caffeine [64].

Interaction of caffeine with *S. cerevisiae* cells was also used for studies on Ribonucleases H [65]. Ribonucleases H are capable of recognizing RNA-DNA duplexes, degrading only the RNA strand, being of high importance in maintaining the genome stability in the eukaryotic cell. RNases H are classified into type 1 and type 2, encoded in yeast by the *RNH1* and *RNH2* genes, respectively [66]. The effects of caffeine were studied in yeast strains carrying deletions of *RNH1*, *RNH2*, or both, and it was noticed that the absence of RNase H1 in a strain that has an active RNase H2 diminishes the deleterious effects of caffeine and that in caffeine-treated cells, the un-degraded RNA-DNA hybrids influence DNA synthesis by damaging or perturbing the cell cycle [67].

## 5. The Target-of-Rapamycin (TOR) Pathway is also the Target-of-Caffeine

Evolutionarily conserved target of rapamycin (TOR) kinase is a major regulator of cell growth and metabolism in response to a broad set of environmental signals and stress conditions. Because its defects were noted to be involved in disorders such as cancer, neurological, metabolic, inflammatory, and autoimmune diseases, as well as in ageing [45,68,69], TOR kinase became a target for many clinical research studies, and investigation of TOR signaling regulators is particularly important for developing effective therapeutic strategies [70,71,72]. The TOR kinase is a member of the phosphatidylinositol 3 (PI3) kinase family, and therefore, it is susceptible to caffeine. In yeast and in higher eukaryotes, the TOR kinase is part of two protein complexes, named TOR Complex 1 (TORC1) and TOR Complex 2 (TORC2) [73].

**Figure 3:**
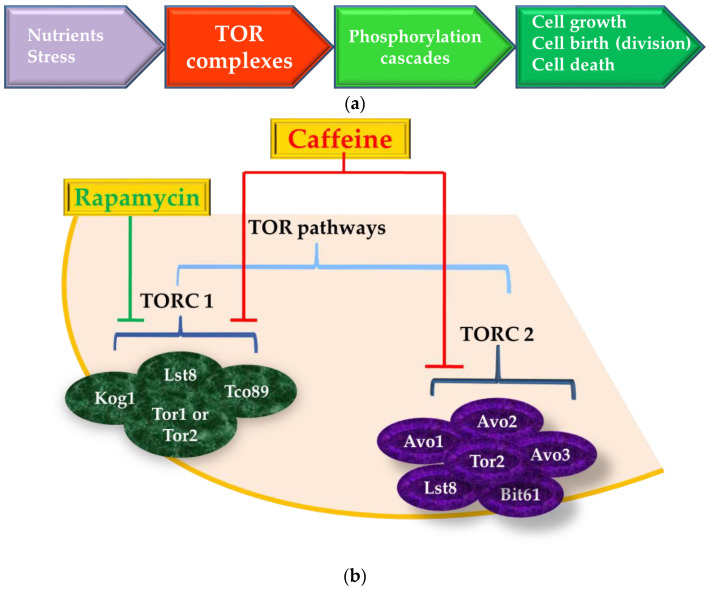
Schematic representation of target of rapamycin (TOR) complexes. (**a**) The main elements up- and downstream of TOR complexes. The TOR complexes are activated by nutrient status or by various stresses. Activated TORCs then initiate phosphorylation cascades involved in regulating fundamental aspects of life such as cell growth, cell birth, and cell death. Adapted after [73,79,80]. (**b**) Caffeine and the TORC in *Saccharomyces cerevisiae* cells. The TOR pathways involve two multiprotein complexes termed TOR complex 1 (TORC1) and TOR complex 2 (TORC2), which are structurally similar but not functionally identical. TORC1 is concentrated at the cell membrane or at the vacuolar membrane and contains Tco89, Lst8, and either Tor1 or Tor2 caffeine-sensitive kinases that act as scaffold to couple TOR and its effectors. The TORC1 is sensitive to rapamicyn. TORC2 is rapamycin insensitive, and it contains Tor2 (but not Tor1) Avo1, Avo2, Avo3, Bit61 (and/or its paralog Bit2), Lst8. TORC2 is found in multiple cellular locations, including the plasma membrane. A plasma membrane location is consistent with the role of TORC2 in controlling the actin cytoskeleton and endocytosis. Adapted after [73,79,80].

The TOR pathways regulate the cellular growth under normal conditions, by stimulating ribosome biogenesis and by controlling the precursors for amino acids and other nitrogenous molecules’ synthesis (Figure 3a). Under harsh environment conditions, such as starvation or excess, the cell metabolic reprogramming is induced via signal transduction pathways involving Tor1 and Tor2, two homologous TOR kinases found in TORC1 and TORC2 [74]. Either Tor1 or Tor2 can function in TORC1, whereas only Tor2 supports TORC2 function (Figure 3b). *S. cerevisiae* has been very useful as model organism for understanding the role of TOR signaling in the regulation of cell growth and aging [75,76], and for this purpose, yeast cells are usually grown under nitrogen starvation or in the presence of inhibitors such as rapamycin [77]. Rapamycin has many natural analogs termed “rapalogs”; one such rapalog is caffeine [78].

The macrolide drug rapamycin is a macrocyclic lactone used as immunosuppressive and anti-proliferative antibiotic which inhibits TORC1 [81]. In the presence of rapamycin, the downstream processes regulated by TORC1 (e.g., stress responses, control of gene expression, protein and ribosome synthesis, amino acid biosynthesis, nitrogen assimilation pathways, protein trafficking and stability, starvation and quiescence, autophagy) are consequently inhibited [82]. While TORC1 is involved in activities related to cell growth, TORC2 is required for polarized cell growth and cytoskeleton organization [73,79]. Rapamycin does not interact with TORC2, nor does it inhibit downstream processes, and therefore, its applications are limited in studying TORC2-related processes [58,64].

In yeast, TORC1 contains kinases Tor1 or Tor2, as well as several additional proteins, including Kog1, Lst8, and Tco89; TORC2 contains Tor2 as well as Lst8, Avo1-Avo3, and Bit61 [79] (Figure 3b). In mammalian cells, TORC1 consists of Tor (mTor), mLST8/GβL (the ortholog of Lst8), and Raptor (the ortholog of Kog1), whereas TORC2 consists of mTor, mLST8/GβL, and mAVO3/Rictor (the ortholog of Avo3p) [81]. Caffeine affects TOR signaling by directly inhibiting TORC1 in many organisms, including yeast, plants, and mammals [83]. It is possible that caffeine also inhibits TORC2 but indirectly, at higher concentrations or upon prolonged treatment [77].

Reinke et al. 2006 [81] were among the first researchers that presented evidence that TORC1 is indeed a significant target for caffeine in yeast by identifying mutations within the FRB (rapamycin binding) and kinase domains of Tor1 that revealed important levels of caffeine resistance that were correlated to highly conserved amino acids within TOR proteins from across the phylogenetic spectrum [81]. Especially in mammals, caffeine was shown to affect cells by direct interaction with components of the TOR pathway [84]. On laboratory animals, rapamycin inhibition of TORC1 leads to delays in ageing, increasing healthy longevity. In human beings, rapamycin is used for preventing organ transplant rejection and to treat some forms of cancer, albeit clinical use is associated with important side effects; this is why the scientific community is in continuous search for TORC1 inhibitors with fewer side effects [85]. Although rapamycin and caffeine induce similar profiles of global gene expression [81,83], it was shown that rapamycin is a partial inhibitor of TORC1 [86], while caffeine is a selective inhibitor of TORC1, acting by a different mechanism from rapamycin [81,87]. Rapamycin binds to the FK506 binding protein FKPB12, and the FKBP12-rapamycin complex inhibits the activity of mTORC1 by destroying the physical interaction between the TOR protein and a second TORC1 component, raptor (Kog1 in yeast) [88,89].

The yeast cells treated with caffeine or rapamycin have a transcriptional profiling that proves the inhibition effect of TOR signaling on a broad array of genes associated with a wide range of cellular growth-related functions and also with stress and autophagy-related genes [83,90]. Notably, similar effects that rapamycin and caffeine display on global gene expression prompted the hypothesis that TOR signaling is mediated through common upstream and downstream regulators, that is, a common intracellular signal transduction pathway, in response to rapamycin and caffeine [83]. The direct target of caffeine in yeast cells is Tor1 kinase, whose inhibition triggers the activation of the Pkc1p-Mpk1p cascade; nevertheless, this activation is not essential for cell survival in the presence of caffeine [13]. In order to investigate if caffeine interferes with the TOR pathway, the transcriptomic responses induced by caffeine and rapamycin were compared [91,92], and it was observed that both compounds trigger down-regulation of the genes involved in transcription, protein synthesis, and ribosome assembly, at the same time activating gene expression in the Krebs cycle, the Gln3p/Gat1p-controlled nitrogen catabolite repression (NCR), and the Rtg1/3p-controlled retrograde pathway [13,91,92]. Gln3 is a major transcription activator that regulates transcription of nitrogen catabolite repression (NCR)-sensitive genes, having high similarity to the DNA binding domain of mammalian GATA factors which induce transcription of target genes [93]. *S. cerevisiae* uses a broad spectrum of compounds as nitrogen sources, and NCR is a physiological response when cells are grown under normal conditions, and preferred nitrogen sources are used (e.g., glutamine) [93]. In cells grown on preferred nitrogen sources, Gln3 is phosphorylated in a TOR-dependent manner, and the transcription of NCR-sensitive genes is repressed. If the cells are grown in the presence of non-preferred nitrogen medium (e.g., proline) or treated with caffeine, Gln3 is dephosphorylated and translocated from the cytoplasm to the nucleus, thereby activating the transcription of NCR-sensitive genes [94]. In this regard, both the intracellular localization and activity of Gln3 are regulated by TORC1 kinase, and caffeine treatment leads to the induction of transcription of NCR-sensitive genes in a similar manner as rapamycin treatment [95].

**Figure 4:**
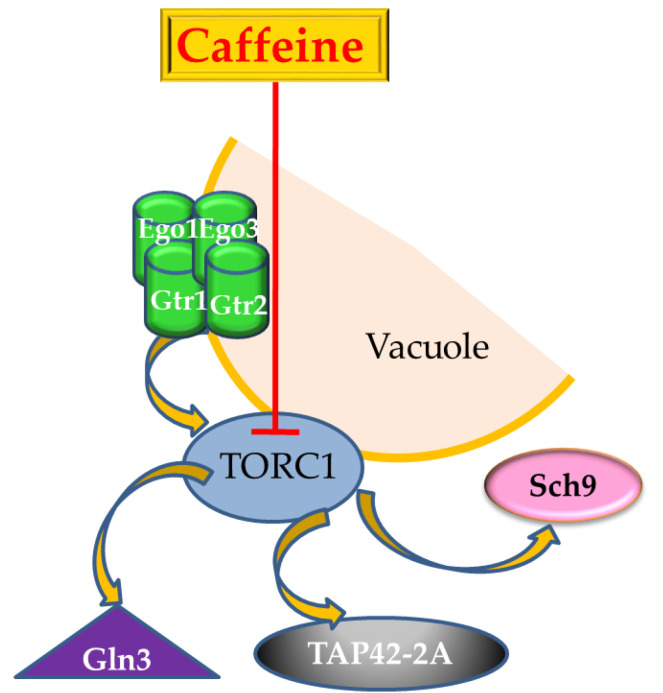
Upstream and downstream components of TORC1 regulation. EGO complex (localized on the vacuolar membrane), which consist in four proteins, Ego1 (a palmitoylated/myristolated protein); Ego3 (a transmembrane protein); and two Ras-family GTPases, Gtr1 and Gtr2, is a major regulator of TORC1 activity, via Tco89. The best characterized substrate of TORC1 is Sch9, a member of AGC family of kinases. When cells are stressed by caffeine, TORC1 is inhibited directly, and the Sch9 phosphorylation is reduced dramatically. TORC1 also regulates the 2A (Pph21, Pph22, and Pph3—generically PP2Ac) and 2A-related phosphatases, including the Tap42-PP2A effector. Inactivation of TORC1 by caffeine results in Tap42 dephosphorylation and TORC1 directly phosphorylate other substrates including Gln3. Adapted from [13,80,81,106].

Rho-family GTPases are key regulators involved in many eukaryotic cell functions (organelle development, cytoskeleton dynamics, cell movement, etc.) and represent a core component of the TORC1 pathway [96,97,98,99,100,101]. In *S. cerevisiae*, the Rho family has six members, Rho1 to Rho5 and Cdc42. Mutants with *RHO5* gene deleted (*rho5*Δ) had a higher resistance to caffeine, in contrast to the Slt2 mitogen activated pathway kinase (MAPK) mutants, which were highly sensitive to caffeine, indicating a role for *RHO5* in the down regulation of the Slt2-MAPK pathway. This special behavior was explained by the fact that Rho5 acts as an off-switch for the MAPK cascade, which differentiates between MAPK-dependent and independent functions of Pkc1, a prototypic member of the protein kinase C superfamily and the main effector of Rho1 [102]. Rho1 is activated by Rom2, its guanine nucleotide exchange factor (GEF), and several integrin-like cell surface proteins, such as Wsc1 and Mid2 [103]. In stress conditions for the cell wall, these cell surface proteins act as stress sensors and activate Rom2 [99,104]. Upon activation, Rho1 binds directly to Kog1, a component of TORC1, leading to a decrease of activity of TORC1. Consequently, the binding also induced dephosphorylation of Gln3 triggering the release and activation of the Tap42-2A phosphatase (Figure 4), a major effector of TORC1 [100,105]. It was demonstrated that caffeine, just like rapamycin, calcofluor white (a cell wall damage agent), nitrogen starvation, and heat, induces Rho1 activation and directly inhibits TORC1, acting both upstream and downstream of Rho1 GTPase [100].

The Ras-family-GTPase and its homologs [107] mediate the growth factor-dependent or stress-induced signal transduction. In yeasts, the Ras-GTPase is a group of enzymes that comprise Ras1, Ypt1, Cdc42, Gtr2, Arf1, Gtr1, and Gsp1; Gtr1 can form a heterodimer with Gtr2 [108]. These proteins switch between an active GTP-bound form and an inactive GDP-bound form and act as molecular switches for various signaling pathways [109]. In yeast, the Gtr1 and Gtr2 (Figure 4) are proteins involved in response to heat shock and in the pathways that are activated during nitrogen starvation and caffeine treatment, suggesting that they have roles in the TOR kinase pathway [110]. It was shown that *Δgtr1* and *Δgtr2* have the similar caffeine-sensitive phenotype [110], in concordance with the previous findings of the inhibitory activity of caffeine on the TOR kinase activity [81,91]. Moreover, Gtr1 and Gtr2 were shown to be involved in the response to oxidative stress and caffeine treatment, acting at Ego1 and Ego3 levels (Figure 4), which genetically interact with components of the TOR signaling pathway [111], the EGO complex being a non-essential activator of TORC1 [80,112]. The guanine nucleotide region of Gtr1p situated at the *N*-terminus is required for Gtr1p–Gtr2p heterodimer formation but not for complex formation with Ego1p, a vacuolar membrane protein. Upon caffeine treatment, the amount of free Gtr1p increases, while it decreases in the protein complexes. Likewise, free Gtr2p is increased by caffeine but the amount form bound in the high molecular weight complexes remains unaffected, indicating that Gtr1p and Gtr2p are necessary for caffeine resistance and that caffeine treatment released Gtr1p from the Gtr1p–Gtr2p complex [110].

In a study over the sensitivity of TORC1 during the rapamycin treatment, it was revealed that the cells recovered efficiently from treatment with saturating concentration of rapamycin alone, as well as with the caffeine alone, and that caffeine is a selective inhibitor of rapamycin-insensitive proliferation; at the same time, the rapamycin-caffeine co-treatment followed by recovery in the presence of caffeine, induced a strong recovery defect [86]. These observations suggested that rapamycin-insensitive TORC1 activity is sensitive to caffeine and is required for residual proliferation rate in the presence of rapamycin and for recovery from the drug [86].

Some new components of TOR signaling were recently identified following direct and specific inhibition of TOR signaling by caffeine and rapamycin using a network-based multi-omics integrative analysis that employed data from transcriptomics, interactomics, and regulomics sources in yeast [80,111,112,113]. The analysis identified seven previously unannotated proteins, Atg14, Rim20, Ret2, Spt21, Ylr257W, Ymr295c, and Ygr017w, as potential components of TOR-mediated rapamycin and caffeine signaling in *S. cerevisiae*. Study of Ylr257w would be particularly informative since it was the only protein whose removal from the constructed network blocked the signal transduction to the TORC1 effector kinase Npr1 [80,111].

A functional link between Ptc1 and the TOR pathway was established due to the rapamycin and caffeine sensitivity of yeast *ptc1* mutants. Ptc1 is a 2C phosphatase isoform, member of the 2C phosphatase family; a connection with TOR pathways is not shared by most members of the family. Ptc1 is required for normal Gln3 and Msn2-mediated transcriptional responses and nuclear localization [91]. In yeast *ptc1* mutants exposed to rapamycin and caffeine, the translocation of Gln3 and Msn2 to the nucleus is prevented and also the dephosphorylation of the Npr1 kinase. At the same time, the overexpression of other isoforms (such as *PTC2* or *PTC3*) did not confer tolerance to rapamycin, and *ptc1 ptc6* double mutant were more sensitive to both rapamycin and caffeine, suggesting the role of both phosphatases in the signaling of TOR pathway [114].

At the level of the general amino acid control (GAAC) and TOR pathways, the cellular stress response is regulated by the amino acylation status of the cellular tRNA pool, which directs the transcriptional regulation of gene expression in response to nutritional stresses [115]. Under normal nutrient conditions, the TOR pathway regulates the cellular growth in a positive manner, by stimulating ribosome biogenesis and utilization of precursors for the synthesis of amino acids and other nitrogenous macromolecules. Under starvation conditions, yeast cells start metabolic reprogramming via signal transduction pathways involving the two homologous protein kinases, Tor1 and Tor2. The cells with a deficient quality control were more tolerant to caffeine than the wild type cells, due to altered interactions between caffeine and the TOR and GAAC pathways components; the increased caffeine tolerance was correlated with a decreased activity of Gln3 [76].

In yeast, twelve lysine methyltransferases that modify translational elongation factors and ribosomal proteins were identified. Among them, five (Efm1, Efm4, Efm5, Efm6, and Efm7) are specific to elongation factor 1A (EF1A), the protein responsible for bringing aminoacyl-tRNAs to the ribosome. It was demonstrated that loss of EF1A methylation is not essential to cell viability but leads to a decrease in growth rates under caffeine and rapamycin treatment. These findings suggested that EF1A interacts with the TORC1 pathway and that Efm methyltransferases are devoted to the modification of EF1A, finding no evidence for the methylation of other substrates in the yeast cell [116].

The mammalian lysosome has an analogue in yeast, the vacuole, a membrane-bounded organelle. The yeast vacuole contains an acidic environment due to vacuolar hydrolases that degrade structural debris macromolecules and waste products [117]. In a genomic screen of 4828 yeasts haploid deletion strains for growth hypersensitivity to hygromycin B (*hhy* mutants), all the *hhy* mutants revealed severe sensitivities to caffeine and rapamycin, suggesting an interaction between the identified genes in TOR kinase pathway [118,119].

## 6. Caffeine and the Yeast Cell Wall Integrity Pathway

Investigations regarding the interaction between caffeine and yeast cells demonstrated the existence of additional caffeine targets, including components of cell wall integrity (CWI) pathways [120]. The cell wall of *S. cerevisiae* confers cell shape and protection against harsh environments [121]. It is formed by different types of molecules, including mannoproteins, glucans, and chitin, closely interconnected. For defense against external insults or for adaptation to cell wall defects, cells use a complex CWI signaling pathways. Inhibition of the synthesis of any structural compounds leads to cell death, making the yeast cell wall an attractive target for antifungal therapy against invasive fungi such as *Candida* spp., *Cryptococcus neoformans*, *Aspergillus* spp., *Pneumocystis carinii,* or *Histoplasma capsulatum* [122]. The CWI involves the MAPK cascade downstream of PKC (protein kinase C) signal transduction pathway [123]. Rho1p GTPase controls the CWI, functions in actin polarization [124], and activates the MAPK pathway [123]. A plethora of studies and biochemical evidence suggested links between TOR and CWI pathways, and caffeine was often used as a phenotypic criterion to evaluate the function of the Mpk1-mediated CWI pathway [123]. In *S. cerevisiae,* it was observed that sensitivity to caffeine can be correlated with defects in the CWI pathway and that caffeine activates CWI signaling, when the stability of the cell wall can be monitored in terms of response to osmotic or thermal stress [123]. It was shown that caffeine is not a typical activator of CWI signaling, because it induces phosphorylation of the Mpk1 *C*-terminus at Ser423 and Ser428 residues independently of the standard dual phosphorylation associated with MAPK activation; nevertheless, these phosphorylations are dependent on the DNA damage checkpoint kinases, Mec1/Tel1 and Rad53 [124]. Other studies also confirmed that yeast strains with altered CWI are caffeine sensitive, including strains lacking one or more of the five *PRS* (phospho ribosylpyrophosphate synthetase) genes, in particular those lacking the Prs1/Prs3 minimal functional unit [125]. In altered versions of *PRS1*, there is a correlation between caffeine sensitivity and increased basal expression of Rlm1, the transcription factor which is an important component of the PKC-mediated MAPK pathway involved in the maintenance of CWI [126].

The loss of function of other proteins involved in CWI can also be related to caffeine sensitive phenotypes. For example, there are six proteins that have the tetratricopeptide repeat (TPR) domain (mediates protein–protein interaction), which are encoded by six essential genes in the *S. cerevisiae* genome. Among these, YNL313c, renamed *EMW1* (essential for the maintenance of the cell wall), proved to be essential for the maintenance of CWI, and the mutants lacking *EMW1* showed sensitivity to diverse stressor compounds, including caffeine [127]. Moreover, the newly described mutant *rim21Δ (ynl294c)* showed a moderate hypersensitivity to caffeine owed to a low compensatory response of the cell wall, indicated by the almost complete absence of Slt2 phosphorylation and the modest increase in chitin synthesis after calcofluor treatment [128].

Cell signaling, gene expression and mitosis but also CWI are cellular processes regulated by phosphorylation/dephosphorylation [129]. Based on sequence analysis of *S. cerevisiae* genes (approximately 6000 genes), the yeast has 117 protein kinase (PKase) and 32 protein phosphatases (PPase) genes [130]. As defects in MAPK pathway are often associated with sensitivity to caffeine, a systematic analysis of caffeine-related phenotype in relation with phosphorylation/dephosphorylation and CWI is still a desiderate [106,130,131].

Caffeine, as a CWI pathway activator, was used to show that Puf5 has a role in response to DNA replication stress and does not involve Pop2. Puf5 is a prototypical PUF protein, a family of RNA binding proteins conserved in eukaryotes, with roles in cell growth, division, differentiation, and development [132]. In *S. cerevisiae* cells treated with caffeine, *PUF5* and *POP2* have the same genetic pathway, leading to the conclusion that the CWI functions are mediated by Puf5 or Pop2-mediated gene repression mechanisms [133].

## 7. Other Pathways Susceptible to Caffeine

Inositol hexakisphosphate (IP6) is the most abundant inositol polyphosphate present in eukaryotes. IP6 is phosphorylated by IP6 kinases (IP6K-s) yielding inositol pyrophosphates, which are important signaling molecules in the eukaryotic cell [134]. Yeast lacking the IP6K known as *Kcs1* display defective vesicular endocytosis, showing a decrease in cell growth [134], sensitivity to environmental stresses [135], and abnormal ribosomal functions [136]. Inositol pyrophosphates are involved in signaling cascades that mediate cell death and telomere length, and they physiologically inhibit signaling by Tel1 and possibly Mec1. Caffeine inhibits the PI3K-related protein kinases Tel1 and Mec1, and therefore, it is expected that *kcs1*Δ mutants are resistant to its lethal effects. Indeed, the lethal action of caffeine is suppressed in mutants that cannot synthesize inositol pyrophosphates because they physiologically antagonize the actions of Tel1 and Mec1 kinases [137].

Other examples of using the pleiotropic action of caffeine on *S. cerevisiae* to understand different molecular mechanism highly conserved in superior eukaryotes and to elucidate the way of action of compounds with potential as human drugs are presented below.

The major component of Lewis Bodies (protein aggregates present in the cytoplasm of neuronal cells in PD (Parkinson Disease)) is the natively disordered protein, α-synuclein [138]. A *S. cerevisiae* proteotoxicity model of PD was employed to evaluate the role of caffeine in the aggregation of α-synuclein. On caffeine treatment, the toxicity of aggregates decreased, the intracellular oxidative stress was diminished, and the survival of the cell increased. It is supposed that caffeine alters the aggregation pathway of α-synuclein by introducing species with reduced proteotoxicity, leading to a decrease of the lag time and an increase in the apparent rate of fibrillation of α-synuclein. α-Synuclein has the ability to assume alternate aggregation pathways more than any other protein that apparently is misfolded in neurodegenerative disorders, because of its natively disordered structure. This effect apparently is heightened by the presence of caffeine, supporting the epidemiological studies that showed that coffee consumption is inversely related to the risk of onset of PD [139,140].

Early studies also introduced caffeine as an activator of the cAMP-dependent protein kinase pathway, based on the in vitro potency of this compound to inhibit the mammalian cAMP phosphodiesterase [141]. In yeast, this hypothesis is still controversial, as some researchers reported an increase of cAMP levels [142], others mentioned no effect on levels of cAMP [143] while other authors showed that caffeine antagonizes the glucose-induced cAMP synthesis [91,144]. Caffeine modifies the metabolic effects produced in the *S. cerevisiae* cell by exposure to glucose, acting on a crossover point at the level of the phosphofructokinase/fructose-bisphosphatase cycle, increasing the ATP levels. Following glucose entry into the cell, caffeine reduces the concentration of intracellular cAMP in a dose-dependent manner, an effect that can be explained by the interference with catabolic inactivation of enzymes [144].

Mitochondria play a fundamental role in eukaryotic cell physiology by integrating numerous death signals, being involved in the control of apoptosis. Mitochondrial genome integrity is essential for the viability of most species. Two mutants of *S. cerevisiae* defective in genes involved in the biosynthesis of mitochondrial phosphatidylglycerol and cardiolipin, *pell* and *crd1*, were analyzed in the presence of different cell wall perturbing agents. The mutants containing dysfunctional mitochondria revealed a modified sensitivity to metabolic inhibitors. The *S. cerevisiae pell* mutant showed increased sensitivity to the cell-wall perturbing agents such as caffeine, caspofungin, and hygromycin [145].

## 8. Caffeine and Lifespan

The ageing biology is a new field that emerged since researchers have been attempting to extent the organisms’ lifespan (LS), and caloric restriction is a critical method used to understand the mechanism of LS. Because caloric restriction is usually accompanied by a reduction in food consumption over a long period of time, chemical food substitutes called caloric restriction mimetics have been the topic of intense research, and they can be used as starting materials in developing drugs that prevent or ameliorate the ageing-associated illnesses. Such compounds have shown their ability to extend the LS in different model organisms, e.g., rapamycin in mice [146] and yeasts [147]. *S. cerevisiae* is an excellent model to study LS as it has conserved ageing pathways, and the study of new molecules’ effect on LS is greatly facilitated by yeast studies which yield significant information before proceeding to animal studies [148].

Physical exercise, caloric restriction, and consumption of moderate amounts of substances such as selenium, zinc, omega 3 unsaturated fatty acids, vitamins E and C, antioxidants, caffeine, or alcohol were proposed as factors essential to extend the human LS or to reduce age-associated diseases. Often, these studies show only correlative (not causative) effects between a compound and longevity. The conservation of most ageing pathways in yeast and their facile genetic manipulation represents a premise to distinguish between the correlative and causative effects of nutrition on ageing [149].

The extent of the LS was studied on *S. cerevisiae* for identifying conserved genetic and pharmacological interventions [150]. The TOR pathway, for example, was first described as genetically involved in aging using experiments made on yeast [151]. Among the many nutraceuticals tested, caffeine was the only compound that induced growth kinetics consistent with a TOR inhibitory effect, increasing doubling time specifically in the *tor1*Δ mutant cells. Studies correlating caffeine, TOR pathway, and LS have since been done on budding yeast [81,87] and fission yeast [83], invertebrate models [152,153] and humans [85,154].

Caffeine treatment of yeast cells releases Rim15 from TORC1-Sch9-mediated inhibition and as a result, it increases LS. Therefore, it is highly probable that an analogous mTORC1/S6K/LATS kinase cascade also has influence on longevity in higher eukaryotes, including humans [155,156]. It was shown that low doses of caffeine significantly extended chronological LS, and partial loss of TORC1 activity increased chronological LS via TORC1–Sch9–Rim15 kinase cascade. Moreover, it was shown that moderate coffee consumption is expected to cause a 4–8% inhibition of mTORC1 activity, suggesting causality explanations for correlation between coffee consumption and longevity [87].

The effect of a polyphenol-rich extract from cocoa on the chronological LS of *S. cerevisiae* was studied under two settings: in the stationary phase reached after glucose depletion and under severe caloric restriction. It was observed that cocoa polyphenol-rich extracts increased the chronological LS of *S. cerevisiae* during the stationary phase in a dose-dependent manner and also extended yeast LS under severe caloric restriction conditions. The cocoa extracts increased the lifespan of wild type cells and also of the *sod2Δ* cells, proving that the mechanism is Mn-SOD2-independent. Nevertheless, this effect was detected only for the polyphenol-rich cocoa extract and not for its individual components, including caffeine [148].

Caffeine (along with curcumin, dapsone, metformin, rapamycin, resveratrol, and spermidine) was evaluated as a LS extender in *S. cerevisiae* under conditions of caloric restriction. In contrast with other studies, caffeine has been claimed to increase the LS of yeast [87], while other groups showed that caffeine, even at higher concentrations, had no effect on LS [157]. Haploid strains of yeast are sometimes unstable in respect to respiratory competence and spontaneously produce the respiration-deficient (RD) mutants with very high frequencies. It was shown that the addition of caffeine to the culture media considerably reduced the production of RD mutants, albeit temporarily [158].

Pathologic endogenous DNA double-strand breaks (EDSB) can occur spontaneously even without exposure to radiation or DNA damaging agents [159]. EDSB can be detected in excess when non-dividing cells have functional DSB repair defects produced independently of replication, a reason why they were named pathologic replication-independent EDSBs (Path-RIND-EDSB) [160]. In chronological aging yeast, reduction of physiologic replication-independent endogenous DNA double strand breaks (Phy-RIND-EDSB) lead to an increase of pathologic RIND-EDSBs (Path-RIND-EDSB); the latter must be repaired instantly as their accumulation can lead to senescence and death [161] or at least a decrease in the cell’s viability [162]. In DSB repair-defective cells, the retention of Path-RIND-EDSBs can occur, a phenomenon that is normally encountered in chronological aging yeast. In caffeine-treated cells, significant accumulation of Path-RIND-EDSB was recorded as quantitatively similar to aging cells with defects in DSB repair, making caffeine an invaluable tool in mimicking chronological aging in vitro [159].

## 9. Concluding Remarks

Caffeine, one of the most consumed and widely accepted neurostimulants, is also a powerful agent used in life science research. Due to its pleiotropic effects [163], caffeine is an active modulator of different enzymes and their regulatory pathways, which include important molecular players such as TOR kinases or DNA damage checkpoint kinases. Many of the studies reviewed here, which made use of the interaction between caffeine and *S. cerevisiae,* contributed to elucidating molecular mechanisms involved in biologic processes of general concern, such as DNA repair mechanisms, cancer, or aging. Using various approaches and setting multiple targets, the studies on caffeine–*S. cerevisiae* interaction generated outputs which could be extrapolated to higher organisms. In spite of the pleiotropic effects of caffeine, there is one mechanism universally accepted, i.e., the inhibitory effect on PI3 kinases, including the core kinases from the TOR complexes. However, since many of the puzzle pieces are still missing, it is no doubt that the duo caffeine–*S. cerevisiae* has not yet reached its full potential in opening doors to new knowledge.
